# The* In Vivo* Dynamics of HIV Infection with the Influence of Cytotoxic T Lymphocyte Cells

**DOI:** 10.1155/2017/2124789

**Published:** 2017-11-14

**Authors:** Purity Ngina, Rachel Waema Mbogo, Livingstone S. Luboobi

**Affiliations:** Institute of Mathematical Sciences, Strathmore University, P.O. Box 59857, Nairobi 00200, Kenya

## Abstract

The* in vivo* dynamics of HIV infection, the infection mechanism, the cell types infected, and the role played by the cytotoxic cells are poorly understood. This paper uses mathematical modelling as a tool to investigate and analyze the immune system dynamics in the presence of HIV infection. We formulate a six-dimensional model of nonlinear ordinary differential equations derived from known biological interaction mechanisms between the immune cells and the HIV virions. The existence and uniqueness as well as positivity and boundedness of the solutions to the differential equations are proved. Furthermore, the disease-free reproduction number is derived and the local asymptotic stability of the model investigated. In addition, numerical analysis is carried out to illustrate the importance of having *R*_0_ < 1. Lastly, the biological dynamics of HIV* in vivo* infection are graphically represented. The results indicate that, at acute infection, the cytotoxic T-cells play a paramount role in reducing HIV viral replication. In addition, the results emphasize the importance of developing controls, interventions, and management policies that when implemented would lead to viral suppression during acute infection.

## 1. Introduction

For the last three decades Human Immunodeficiency Virus (HIV) has been a big challenge in the whole world though its impact is mostly felt in Sub-Saharan Africa. Over 36 million people have been infected since early 1980s and over 25 million of died [1]. Due to the high mortality associated with the virus, HIV has become a major problem for human Health. Many researchers [2–5] have sought to analyze the infection mechanism of the virus. It has been found that HIV targets and infects CD4^+^ T-cells. This is because CD4^+^ T-cells have a protein on its surface that can bind to foreign substances such as HIV, that is, through exploitation of the CCR5 and CXCR4 coreceptors expressed on their surfaces. Once inside the CD4^+^ T-cells, the HIV, which is a retrovirus, is converted to DNA. The virus then multiplies inside the cells that burst releasing more mature virions. This in turn triggers the thymus to produce more CD4^+^ T-cells. Consequently, more HIV virions are produced. Hence, the major hallmarks of HIV infection include the destruction of helper CD4^+^ T lymphocytes and subsequent loss of immune competence. HIV virions in particular weakens the cell function by damaging the helper cells necessary in building a robust immune response. Depletion of the CD4^+^ T-cells results in a weakened immune system [6].

During the initial infection stage, high level of viral replication takes place lasting for about three to six weeks upon infection [3]. This period is followed by the asymptomatic stage which is characterized by high level of immune response; this helps in stabilizing the viral load in the infected person. This stage lasts for several years and varies from patient to patient. It is important to note that during this period the infected person shows no sign of the infection. However, if not treated the virus may progress to disease/symptomatic/AIDS stage. This is when the body is prone to many opportunistic infections. It is characterized by a decrease in the number of CD4^+^ T-cells and an increase in the viral load. In addition, within-host virus genetic diversity decreases [7, 8].

Mathematicians in the field of epidemiological modelling have developed models to analyze the HIV infection mechanism* in vivo*. These models have provided important insights into diseases behaviors and how best it could be controlled. To date, mathematical modelling has become a paramount tool, in understanding the dynamics of HIV and in decision making processes regarding intervention programmes for controlling and managing the virus in many countries. Arruda et al. [9] proposed and analyzed a five-dimensional model for HIV infection* in vivo* with the inclusion of the CD8^+^ T-cells. As much as the study included the activation process in the* in vivo* model the argument that the CD8^+^ T-cells kills the virus directly is clinically wrong. Hattaf and Yousfi [10] analyzed an* in vivo* HIV model. However, the model only included the CD4^+^ T-cells and the virus and omitted the infected CD4^+^ T-cells. Ogunlaran and Oukouomi Noutchie [11] analyzed a three-dimensional model, which included the CD4^+^ T-cells, the infected CD4^+^ T-cells, and the HIV virions. This study was more interested in establishing how to maximize the number of the infected cells after introduction of ARTs. Nonetheless, the study failed to put in account the role played by the CD8^+^ T-cells in fighting the virions. Omission of such important variables in the model paints the wrong picture of the disease dynamics. The question of the role played by one's immune system could not be answered by such a model.

Zarei et al. [12] developed a five-dimensional* in vivo* HIV model. The study included concentration of healthy CD4^+^ T-cells, concentration of infected CD4^+^ T-cells, and cytotoxic T-cells which were divided into precursors CTLp and effectors and the free virus particles. This study assumed that cytotoxic T lymphocyte (CTL) response depends on CD4^+^ T-cell help and that HIV virions impairs T-helper cell function. Consequently, the proliferation of the CTLp population is proportional to both infected cells in the body and the number of uninfected T-helper cells. The simulated results indicated the importance of the CTL cells in the HIV model. The study had some few shortcomings since it failed to account for the resistant and the wild type CD4^+^ T-cells. The rate in which the two types of the CD4^+^ T-cells are infected by the HIV virions is quite different, hence the need to include them in the model.

Zhuang and Zhu [13] analyzed a three-dimensional in-host HIV model. As much as this model was so basic since it had only three compartments it brought out important insight as far as HIV dynamics are concerned. The time lag from infection of the CD4^+^ T-cells to the cells becoming actively infected was included in the model. The consideration of such a parameter is very important in HIV research. The study established the global existence of bifurcating periodic solutions with the assistance of global Hopf bifurcation theory. The numerical results in the study indicated that the latent period plays an important role in the disease spread and the disease may be controlled by shortening it.

Ngina et al. [3] analyzed a five-dimensional in-host model. The results from the study established the importance of the CD8^+^ T-cells in controlling HIV viral progression. The stability analysis of the model indicated the presence of backward bifurcation implying that having *R*_0_ < 1 does not guarantee eradication of the virus in the body.

This study wishes to improve the research by Ngina et al. [3] by introducing the wild type and and the resistant CD4^+^ T-cells. The study will also be aimed at addressing the gaps noted from the cited literature.

## 2. Model Description

We shall put into consideration a mathematical model for the* in vivo* interaction of the HIV virions and the immune system cells. The model is classified into six compartments. The following are the variables used in the model: the wild type healthy CD4^+^ T-cells (*T*_*w*_); resistant type healthy CD4^+^ T-cells (*T*_*r*_); the infectious HIV virus particles (*V*); the already infected CD4^+^ T-cells (*I*); and the cytotoxic T-cells (CTL), that is, CD8^+^ T-cells (*Z*) and the activated cytotoxic T-cells (*Z*_*a*_).

The wild type healthy CD4^+^ T-cells are recruited at a constant rate *λ*_*T*_*w*__ from the thymus and die naturally at a constant rate *μ*_*T*_*w*__. These cells are infected by the virus at the rate *χ*_*w*_*T*_*w*_*V*. The resistant type healthy CD4^+^ T-cells are recruited from the thymus at a constant rate *λ*_*T*_*r*__ and die naturally at a constant rate *μ*_*T*_*r*__. These cells are infected by the virus at the rate *χ*_*r*_*T*_*r*_*V*. The infected CD4^+^ T-cells result from the infection of both the wild and resistant type healthy CD4^+^ T-cells and die at a rate *μ*_*I*_*I*, and are killed by activated cytotoxic T-cells at the rate *αIZ*_*a*_. They could also be recruited directly from the thymus at a constant rate *λ*_*Z*_. Clinical finding indicates that CTL response depends on infected CD4^+^ T-cells. Consequently, the recruitment into the population of the CTL cells is given by *cZI*. This results from the stimulation by the viral antigen of the infected cells. CTLs are activated at the rate *βZI*. Due to the absence of the viral antigen the CTL T-cells die at the rate *μ*_*Z*_*Z* while the activated CTL cells die at the rate *μ*_*Z*_*a*__*Z*_*a*_. The free HIV virions are produced by the infected CD4^+^ T-cells at the rate *ϵ*_*V*_*μ*_*I*_*I* and they die at the rate *μ*_*V*_*V*.

(1) dTwdt=λTw−μTwTw−χwTwV,dTrdt=λTr−μTrTw−χrTrV,dIdt=VχwTw+χrTr−μII−αIZa,dVdt=ϵVμII−μVV,dZdt=λZ+cZI−μZZ−βZI,dZadt=βZI−μZaZa.

The parameters used in model (1) are described in Table 1

## 3. Properties of the HIV Model

### 3.1. Positivity of Solutions

The* in vivo* HIV model monitors cell population. Hence, there is a need to prove that the state variables for model (1) remain nonnegative. In particular we show that, with nonnegative initial conditions, the solutions of model (1) will remain nonnegative for all time values *t* ≥ 0. We thus have the following theorem.

**Theorem 1 .**

Let the parameters for model (1) be nonnegative constants. A nonnegative solution (*T*_*w*_(*t*), *T*_*r*_(*t*), *I*(*t*), *V*(*t*), *Z*(*t*), *Z*_*a*_(*t*)) for model (1) exists for all state variable with nonnegative initial conditions (*T*_*w*_(0) ≥ 0, *T*_*r*_ ≥ 0, *I*(0) ≥ 0, *V*(0) ≥ 0, *Z*(0) ≥ 0, *Z*_*a*_ ≥ 0) for all *t* ≥ 0.

**Proof**

From the first equation of model (1) we have

(2) dTwdt=λTw−μTwTw−χwTwV=λTw−μTw+χwVTw>−μTw+χwVTw.

By separation of variable method we have

(3) $$\begin{array}{c} \frac{dT_{w}}{T_{w}}>-\left(\;\mu_{T_{w}}+\chi_{w}V\;\right)dt. \end{array}$$

Integrating (3) we have

(4) $$\begin{array}{c} T_{w}>Ce^{-\int_{0}^{t}\left(\mu_{T_{w}}+\chi_{w}V\left(s\right)\right)ds}. \end{array}$$

Taking the initial conditions at *t* = 0 and *T*_*w*_(0) = *T*_*w*_0__ then from (4) we have

(5) $$\begin{array}{c} C=T_{w_{0}}. \end{array}$$

Therefore, (4) can be written as

(6) $$\begin{array}{c} T_{w}>T_{w_{0}}e^{-\int_{0}^{t}\left(\mu_{T_{w}}+\chi_{w}V\left(s\right)\right)ds}. \end{array}$$

Thus,

(7) $$\begin{array}{c} T_{w}\left(\;t\;\right)>0\;\forall t\geq 0. \end{array}$$

Similarly, using the same argument, it can be shown that the state variables *T*_*r*_(*t*) > 0, *I*(*t*) > 0, *V*(*t*) > 0, *Z*(*t*) > 0, *Z*_*a*_(*t*) > 0 are nonnegative for all *t* > 0. Therefore, the solutions of system (1) remain positive for all *t* ≥ 0. This completes the proof.

### 3.2. Boundedness of Solutions

**Theorem 2 .**

All solutions (*T*_*w*_(*t*), *T*_*r*_(*t*), *I*(*t*), *Z*(*t*), *Z*_*a*_(*t*)) ∈ *ℝ*^6^ of model (1) are bounded and there exists a biological feasible region Γ for model (1) given as

(8) Γ=Twt,Tr,It,Vt,Zt,Zat∈R6 ∣ Twt>0,  Trt>0,  It>0,  Vt>0,  Zt>0,  Zat>0.

**Proof**

The total population of the CD4^+^ T-cells, *T*_*w*_ + *T*_*r*_ + *I* = *N*_4_(*t*), is a nonconstant value. Hence, according to (1), the evolution equation representing the change in the population of the CD4^+^ T-cells is given by

(9) dN4tdt=λTw+λTr−μTwTw−μTrTr−μI+αZaI,dN4tdt≤λTw+λTr−μTwTw−μTrTr−μII,dN4tdt≤λTw+λTr−μTw+μTr+μIN4t.

We solve (9) using the separation of variable method for solving differential inequality.

(10) $$\begin{array}{c} \frac{dN_{4}\left(t\right)}{dt}+\left(\;\mu_{T_{w}}+\mu_{T_{r}}+\mu_{I}\;\right)N_{4}\left(\;t\;\right)\leq \lambda_{T_{w}}+\lambda_{T_{r}}. \end{array}$$

Integrating factor for (10) is given by

(11) $$\begin{array}{c} I.F=e^{\left(\mu_{T_{w}}+\mu_{T_{r}}+\mu_{I}\right)t}. \end{array}$$

Multiplying (10) by the integrating factor given in (11) we have,

(12) $$\begin{array}{c} N_{4}\left(\;t\;\right)e^{\left(\mu_{T_{w}}+\mu_{T_{r}}+\mu_{I}\right)t}\leq \frac{\lambda_{T_{w}}+\lambda_{T_{r}}}{\mu_{T_{w}}+\mu_{T_{r}}+\mu_{I}}e^{\left(\mu_{T_{w}}+\mu_{T_{r}}+\mu_{I}\right)t}+C. \end{array}$$

Applying the initial condition in (12), at *t* = 0, and letting *N*(0) = *N*_4_0__, we obtain

(13) $$\begin{array}{c} N_{4_{0}}=\frac{\lambda_{T_{w}}+\lambda_{T_{r}}}{\mu_{T_{w}}+\mu_{T_{r}}+\mu_{I}}+C. \end{array}$$

Hence,

(14) $$\begin{array}{c} C=N_{4_{0}}-\frac{\lambda_{T_{w}}+\lambda_{T_{r}}}{\mu_{T_{w}}+\mu_{T_{r}}+\mu_{I}} \end{array}$$

Substituting (14) into (12) we have

(15) N4t≤e−μTw+μTr+μItλTw+λTrμTw+μTr+μIeμTw+μTr+μIt+N40−λTw+λTrμTw+μTr+μI,N4t≤λTw+λTrμTw+μTr+μI+N40−λTw+λTrμTw+μTr+μI·e−μTw+μTr+μIt

As *t* → *∞* (15) becomes

(16) $$\begin{array}{c} \underset{t\to \infty }{\lim}N_{4}\left(\;t\;\right)\leq \frac{\lambda_{T_{w}}+\lambda_{T_{r}}}{\mu_{T_{w}}+\mu_{T_{r}}+\mu_{I}}. \end{array}$$

Similarly as *t* → 0 (15) becomes

(17) $$\begin{array}{c} \underset{t\to 0}{\lim\;}N_{4}\left(\;t\;\right)\leq N_{4_{0}}. \end{array}$$

From (16) and (17) we conclude that *N*_4_(*t*) is bounded above by

(18) $$\begin{array}{c} N_{4}\left(\;t\;\right)\leq \max\left\{\;N_{4_{0}},Q\;\right\}, \end{array}$$

where *Q* = (*λ*_*T*_*w*__ + *λ*_*T*_*r*__)/(*μ*_*T*_*w*__ + *μ*_*T*_*r*__ + *μ*_*I*_).

From (18) the state variables describing the evolution of the total population of the CD4^+^ T-cells are less than or equal to the ratio of the recruitment rate and the decay rate.

The same procedure can be used to show that all the state variables are bounded. Since all state variables are positive and bounded in *ℝ*^6^, then the region Γ is positively invariant.

*Remark  3*. The biologically feasible region Γ for model (1) defined by the compact set

(19) Γ=Twt,Tr,It,Vt,Zt,Zat∈R6,  Tw+Tr+I≤λTw+λTrμTw+μTr,  Z+Za≤λZμZ,  V≤εVμIλTμTμV+V0

with initial conditions *T*_*w*_(0), *T*_*r*_(0), *I*(0), *V*(0), *Z*(0), *Z*_*a*_(0) > 0 is positively invariant and attracting for all *t* > 0. The domain Γ is positively invariant under the flow induced by the system (1). Therefore, system (1) is biologically meaningful and it is feasible to analyse the model in the domain Γ.

## 4. Disease-Free Equilibrium and Its Stability

The disease-free equilibrium point occurs when there is no infection in the body and hence it is obtained by setting infectious classes in (1) to zero; that is, *V* = *I* = *Z*_*a*_ = 0,

(20) E0=Tw0,Tr0,0,0,Z0,0=λTwμTw,λTrμTr,0,0,λZμZ,0.

### 4.1. Basic Reproductive Number

We apply the next generation matrix method for the derivation of *R*_0_ [16]. *R*_0_ is given by *R*_0_ = *ρ*(*FV*^−1^), where *ρ* is defined as the spectral radius of the next generation matrix [17] and *F* represents the appearance of new infections while *V* is the rate of transfer of the infections [18]. Using the Van den Driessche and Watmough [18] method we have three infection classes, that is, *I*(*t*), *V*(*t*), and *Z*(*t*), and hence the matrix of new infections is given by

(21) $$\begin{array}{c} F=\left[\begin{array}{cccc} 0 & \frac{\chi_{w}\lambda_{T_{w}}}{\mu_{T_{w}}}+\frac{\chi_{r}\lambda_{T_{r}}}{\mu_{T_{r}}} & 0 & \\ 0 & 0 & 0 & \\ c\frac{\lambda_{Z}}{\mu_{Z}} & 0 & 0 & \end{array}\right]. \end{array}$$

The matrix that represents the transfer of the infections between compartments at the disease-free equilibrium is given by

(22) $$\begin{array}{c} V=\left[\begin{array}{ccc} \mu_{I} & 0 & 0\\ -\epsilon_{V}\mu_{I} & \mu_{V} & 0\\ \beta \frac{\lambda_{Z}}{\mu_{Z}} & 0 & \mu_{Z} \end{array}\right]. \end{array}$$

The inverse of *V*^−1^ is given by

(23) $$\begin{array}{c} V^{-1}=\left[\begin{array}{ccc} \frac{1}{\mu_{I}} & 0 & 0\\ \frac{\epsilon_{V}}{\mu_{V}} & \frac{1}{\mu_{V}} & 0\\ -\beta \frac{\lambda_{Z}}{\mu_{I}\mu_{Z}^{2}} & 0 & \frac{1}{\mu_{Z}} \end{array}\right]. \end{array}$$

The next generation matrix *FV*^−1^ is given by

(24) FV−1=χwλTwμTw+χrλTrμTrϵVμVχwλTwμTr+χrλTrμTr1μV0000cλZμZ1μI00.

The eigenvalues for the matrix given by (24) are 0, 0 and ((*λ*_*w*_*μ*_*T*_*r*__*χ*_*T*_*w*__ + *λ*_*T*_*r*__*μ*_*T*_*w*__*χ*_*T*_*r*__)/*μ*_*T*_*r*__*μ*_*T*_*w*__*μ*_*V*_)*ϵ*_*V*_.

Thus the reproductive number *R*_0_, which is given by the greatest eigenvalue, is

(25) R0=λwμTrχTw+λTrμTwχTrμTrμTwμVϵV=λTwχTwμTw+λTrχTrμTrϵVμV.

*R*_0_ as given by (25) represents the number of secondary infection that results from a single infected cell over its average life time 1/*μ*_*V*_. In addition, it is important to note that the infection will die out if *R*_0_ < 1 while the HIV infection may become endemic if *R*_0_ > 1.

### 4.2. Sensitivity Analysis of *R*_0_ with respect to the Model Parameters

The aim of researchers especially in the field of HIV modelling is to understand the dynamics of HIV so as to control it. This is mainly done by targeting some parameters to which *R*_0_ is sensitive.

We apply the normalized forward index method in the analysis. The normalized forward sensitivity index of *R*_0_ with respect to the parameter *P* is given by

(26) $$\begin{array}{c} \frac{\partial R_{0}}{\partial P}*\frac{P}{R_{0}}, \end{array}$$

where *P* represents the parameters on the basic reproductive number. From the basic reproductive number given by (25) we get

(27) ∂R0∂λTwλTwR0=χTwλTwμTrλTwχTWμTr+λTrχTrμTw,∂R0∂λTrλTrR0=χTrλTrμTwλTwχTWμTr+λTrχTrμTw,∂R0∂χTwχTwR0=χTwλTwμTrλTwχTWμTr+λTrχTrμTw,∂R0∂χTrχTrR0=χTrλTrμTwλTwχTWμTr+λTrχTrμTw,∂R0∂μTwμTwR0=−χTwλTwμTrλTwχTWμTr+λTrχTrμTw,∂R0∂μTrμTrR0=−χTrλTrμTwλTwχTWμTr+λTrχTrμTw,∂R0∂ϵVϵVR0=1,∂R0∂μVμVR0=−1.

From the sensitivity index represented as in Table 2 it is evident that *ϵ*_*V*_ is the most positively sensitive parameter. This means that to maintain a small number on *R*_0_ we have to reduce this parameter whereas increasing these parameters will lead to an increase in the *R*_0_ whereas *μ*_*V*_ is the most negatively sensitive parameter. This means that increasing this parameter will decrease the value of *R*_0_.

**Figure 1:**
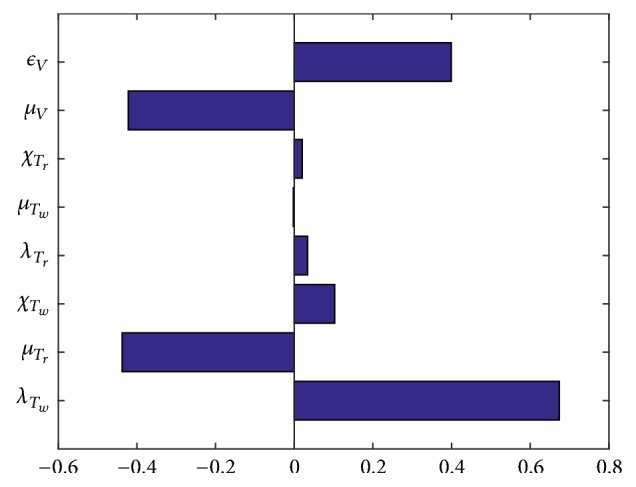
Tornado plot showing the sensitivity of *R*_0_ to some of the parameters values.

Using the parameters values in Table 3 we present the Tornado plots of partial rank correlation coefficients (PRCCs) of the parameters that influence *R*_0_ in Figure 1.

From Figure 1 it is evident that a decrease in the rate of HIV virions production (*ϵ*_*V*_) would lead to a decrease in the value of *R*_0_. This can be done by introducing HIV drugs such as the reverse transcriptase inhibitors (RTI) or the protease inhibitor (PI). RTI prevents the production of more HIV virions since it inhibits the reverse transcription process. If the HIV RNA is not reverse transcribed to HIV DNA, then the virus inside the cells cannot multiply. In addition, use of PIs inhibits the production of protease enzyme that is necessary for the maturation of the HIV virions; consequently, the virus produced after its introduction would be noninfectious and immature. Furthermore, a strong immune response would lead to a reduction in the number of the HIV virions. Activated cytotoxic T-cells fight and kill/remove the infected cells. This in turn reduces the number of the HIV virions produced.

Increase in the death rate of free HIV virions would also lead to a decrease in *R*_0_. This could be done by introducing the ARTs drugs aforementioned. However, it is important for researchers to establish the most optimal HIV drugs that would lead to immune reconstruction with minimal side effects.

### 4.3. Effect of *R*_0_ on the* In Vivo* Dynamics of HIV

In this subsection we establish the effects of *R*_0_ on the dynamics of infected cells and the HIV virions. We apply the parameter values described in Table 3.

**Figure 2:**
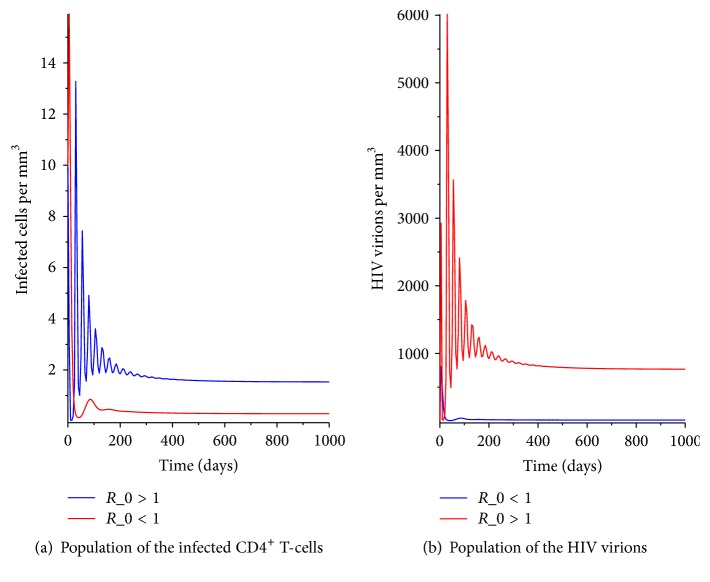
The HIV dynamics with varying *R*_0_.

From Figure 2 it is evident that change in *R*_0_ has a big impact on the magnitude of infected cells and the HIV viral load. It is evident from the graphs in Figure 2 that an increase in *R*_0_; that is, having *R*_0_ > 1 leads to an increase in the number of the HIV virions and the infected cells. This implies that the body immunity is threatened and the infected person may progress to AIDS stage if not treated with the ARTs. However, when *R*_0_ < 1 the number of the HIV virions in the blood reduces significantly; therefore the infection may die out. For medical practitioners to reduce the effect of the infection it is important to ensure that *R*_0_ < 1. This is by developing control, interventions, and management policies that if implemented would ensure that *R*_0_ < 1. In the next section we analyze the stability of the disease-free equilibrium point.

### 4.4. Local Stability of the Disease-Free Equilibrium (DFE)

**Theorem 4 .**

The disease-free equilibrium *E*_0_ of system (1) is locally asymptotically stable if *R*_0_ < 1 and unstable if *R*_0_ > 1.

**Proof**

Van den Driessche and Watmough [18] indicated that the stability of the disease-free equilibrium point of a dynamical system is determined by the stability of the matrix *F* − *V* given by

(28) $$\begin{array}{c} F-V=\left[\begin{array}{cccc} -\mu_{I} & \frac{\chi_{w}\lambda_{T_{w}}}{\mu_{T_{w}}}+\frac{\chi_{r}\lambda_{T_{r}}}{\mu_{T_{r}}} & 0 & \\ \epsilon_{V}\mu_{I} & -\mu_{V} & 0 & \\ \frac{\lambda_{Z}}{\mu_{Z}}\left(c-\beta \right) & 0 & -\mu_{Z} & \end{array}\right]. \end{array}$$

We solve the following to obtain the eigenvalues of (28):

(29) F−V−ΛI=−μI−ΛχwλTwμTw+χrλTrμTr0ϵVμI−μV−Λ0λZμZc−β0−μZ−Λ=0.

The characteristic equation of (28) is given by

(30) $$\begin{array}{c} \Lambda^{3}+b_{2}\Lambda^{2}+b_{1}\Lambda +b_{0}=0, \end{array}$$

where

(31) b2=μZ+μV+μI,b1=μVμZμTrμTw+μVμTrμTwμI+μZμTrμTwμI−μTrχwμTwλTwϵV−μTwχrμTwλTrϵVμTwμTr,b0=μZμ1μVμTrμTw−μTrχwλTwϵV−μTwχrλTrϵVμTwμTr.

Using the Routh-Hurwitz criterion [19], to determine the conditions for the real part of the roots of the characteristic equation (30) Re(Λ) < 0 for 3rd-degree polynomial we require

(32) b2>0,b0>0,b2b1−b0>0.

We can clearly observe from (31) that all the Routh-Hurwitz conditions are satisfied. Thus all the eigenvalues according to the characteristic equation are negative and real. This implies that the virions-free equilibrium point is locally asymptotically stable when *R*_0_ < 1 and unstable when *R*_0_ > 1. The epidemiological implication of Theorem 4 is that the HIV virions could be cleared from the body if and only if *R*_0_ < 1.

## 5. Numerical Simulation

This section is aimed at investigating numerically the behavior of each compartment on the onset of infection without any medical treatment. We use Maple software to analyze the HIV dynamics* in vivo* without any interventions apart from the body immunity. The initial values of the model were set as *T*_*w*__0_ = 1000, *T*_*r*__0_ = 10, *I*_0_ = 10, *V*_0_ = 10, *Z*_0_ = 500, and *Z*_*a*__0_ = 30. The values for the parameter are adopted from Table 3.

**Figure 3:**
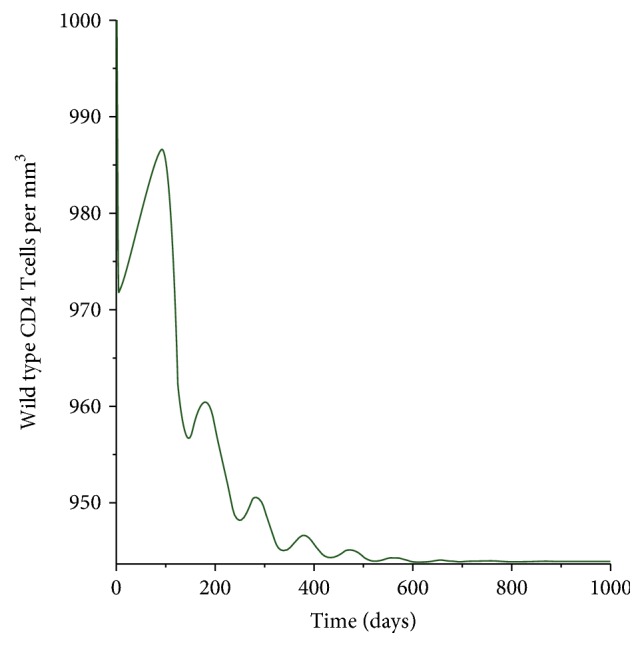
Population of the wild type CD4^+^ T-cells.

Figure 3 shows the dynamics of the wild type CD4^+^ T-cells. The hallmark of HIV/AIDS pathogenesis is the depletion of CD4^+^ T-cell populations. It is evident from Figure 3 that as the disease progresses the number of the CD4^+^ T-cells decreases. However, due to the immune system mechanism the reduction of the CD4^+^ T-cells is followed by an increase in the number of the CD4^*T*^ which coincides with immune system reconstruction. This can be explained by the fact that the body mechanism will always try to be at an equilibrium. However, as the immune system weakens the body is unable to reconstruct itself, and that is why we get a straight line after the second year. The results in this study agrees with clinical observation [20–22]. It has been indicated that initial destruction of the cells is counteracted by CD4^+^ memory T-cell regeneration that preserves CD4+ T-cell numbers. The number, however, does not go back to preinfection stage. This process is not maintained for a longer period and that is why we see a drastic drop in the level of the CD4^+^ T-cells. In HIV as the number of the cells decreases the body immunity lacks the ability to fight other infections. That is why HIV infected people are prone to many opportunistic deceases as the CD^+^ T-cells go below 350 cells/mm^3^.

**Figure 4:**
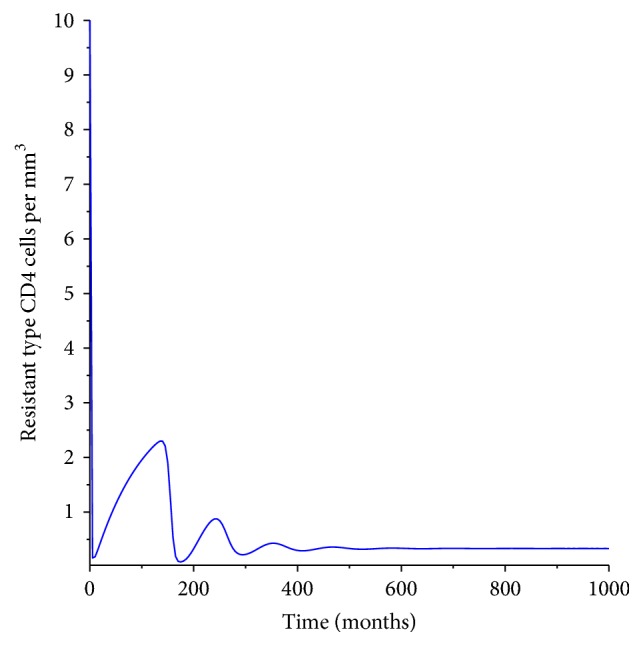
Population of the resistant type CD4^+^ T-cells.

Figure 4 shows the dynamic of the resistant CD4^+^ T-cells. The dynamics of these cells are similar to that of the wild type cells. Nonetheless since the cells resist infection they remain at a low level after the third month, which is not the case with the wild type CD4^+^ T-cells.

**Figure 5:**
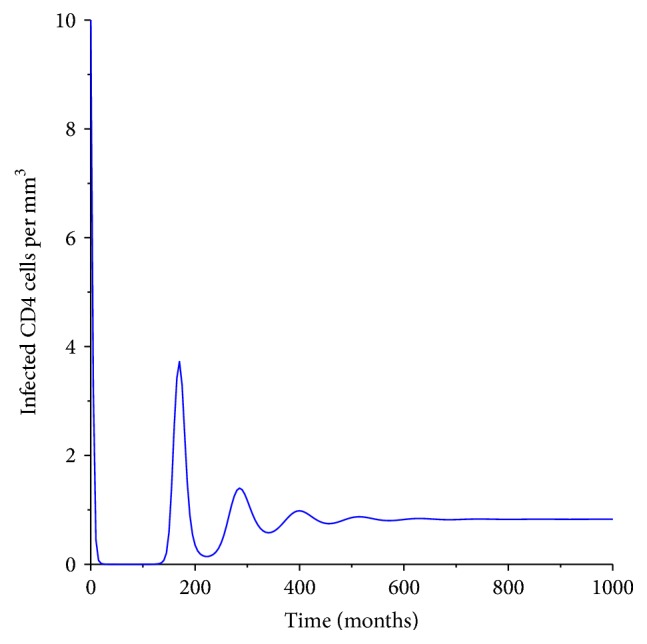
Population of the infected CD4^+^ T-cells.

Figure 5 shows the dynamics of the infected CD4^+^ T-cells. It is evident that at acute infection the number of the infected cells increases at a very sharp rate and then decreases exponentially. However, after the 100 days the level increases, but since the body has a way of balancing the cells, we see an increase is followed by a decrease. The harmonic oscillations is maintained up to 500 days. Due to the weak immune system the number of infected cells remains at a constant rate from 600 days which might remain so for several years. HIV has proved to have no cure so far. However, as researchers we need to find a way of killing all the infected cells before they bud out and produce mature HIV virions. So far clinicians have indicated that HIV-induced cell death actually increases HIV replication [23, 24].

**Figure 6:**
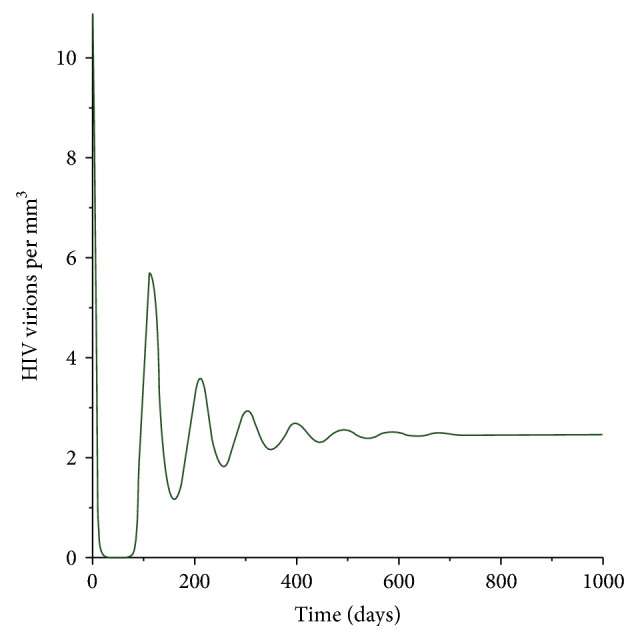
Population of the HIV virus particles.

Figure 6 represents the dynamics of the HIV virions for the first 1000 days after infection. It is evident that the number of HIV virions increases in the first few days after infection. Afterwards the number of virions decreases. This is because of the recruitment of the cytotoxic cells to fight the free virus. After about three months the level increases exponentially; this is because many infected cells burst releasing a higher number of the virions. Since the cytotoxic cells kill the infected cells then indirectly they reduce the number of HIV virions produced. A sharp increase after three months is, therefore, followed by a decrease in the number of HIV virions. After 500 days, the number of HIV virions remains at a constant rate. It is important to note that new HIV virions are emitted from an infected CD4^+^ T-cell, via bursting of the cell. This implies that a single burst produces a big number of new HIV particles.

**Figure 7:**
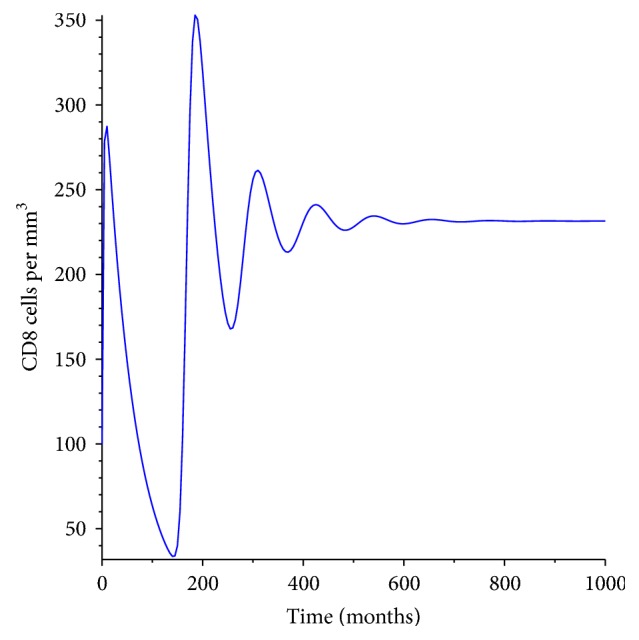
Population of the cytotoxic T-cells.

**Figure 8:**
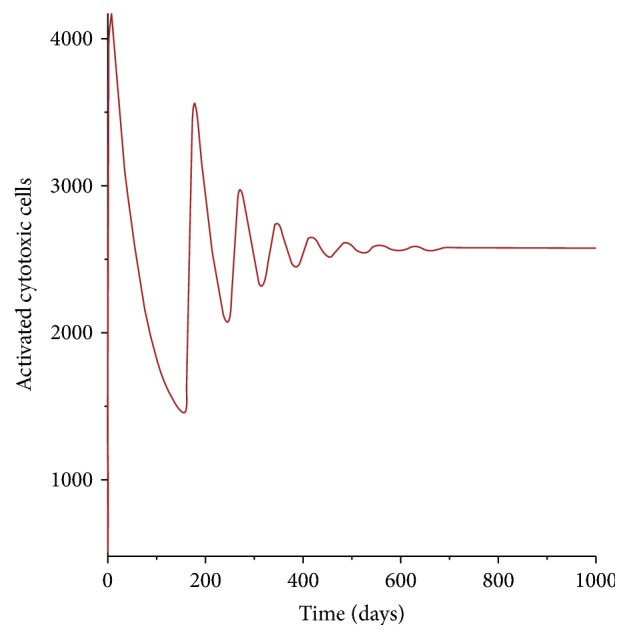
Population of the activated cytotoxic T-cells.

Figure 7 represents the dynamics of the of the cytotoxic cells in the first 1000 days after infection. These are specialized cells of the adaptive immune system capable of finding and eliminating pathogen-infected cells. They are responsible for destroying and killing the infected cells and in turn help to restore the immune system. They arise from the bone marrow and later relocate to the thymus for maturation. During this process they are able to express a unique antigen-binding molecule known as the T-cell receptor. The receptor enables them to monitor all cells of the body, ready to destroy any cell posing a threat to the organism. Nonetheless, for the cytotoxic cells to fight and destroy any infected cell they must be activated and the dynamics of the activated cells is shown in Figure 8. The activation takes place at the surface of accessory cells, which mature during the innate immune responses triggered by an infection.

From Figure 8 it is evident that the number of the activated CD8^+^ T-cells increases exponentially for the first month. The cells are activated in preparation to kill the already infected CD4^+^ T-cells. The number then reduces to a minimum after 5 months (150 days) though not to the level of the preinfection period. This coincides with the reduction in the number of the free HIV virions. Onward a nonharmonic curve is seen for the dynamics of the activated cytotoxic cells.

## 6. Conclusion

This paper presented a six-dimensional* in vivo* HIV dynamics model. The model analyzes HIV virus dynamics focusing on the highly dynamic interaction between HIV virions, uninfected wild and resistant type CD4^+^ T-cells, infected CD4^+^ T-cells, and CTLs. The inclusion of the immune response to viral infection was a key feature in examining the course of HIV infection. The model was aimed at analyzing the mechanism of the HIV virus during the entry time up to the maturation time and the role played by the activated CD8^+^ T-cells in fighting and killing the HIV virions. We started by proving that the model was epidemiologically well posed. We later derived the expression of the basic reproductive number, *R*_0_. It was evident that the model was locally stable and the simulated results from the model emphasized the importance of maintaining *R*_0_ below one. The cytotoxic cells play a very crucial role in our system as far as infection control is concerned. It is evident from the numerical results that high level of the virus and infected cells in the body result in an increase in the level of the activated defense cells. The activated cells fight the infected cells and indirectly reduce the viral load. In addition, due to the high increase of the virions during the first three months it is important to introduce ARTs to prevent HIV transmission. This will help in the reduction of new infection. From this study we note that there is production of high number of HIV virions during the early stages of infection; it is therefore paramount to initiate ARTs to prevent HIV transmission. In addition, the medical practitioners and the government should initiate HIV programs and management polices that will lead to having *R*_0_ < 1.

In conclusion, lessons learnt by the various researchers, governmental and nongovernmental organizations, and clinicians in addressing the HIV for the last three decades must be collaboratively collected and the findings implemented. In future it is important to carryout the optimal control to establish the role played by the HIV drugs and also the optimal drug combinations.

## Figures and Tables

**Table 1:** Parameters for *in vivo* HIV dynamics with therapy model.

Parameter	Description
*λ*_*T*_*w*__	The rate at which the wild type non-infected CD4^+^ T-cells are produced.
*λ*_*T*_*r*__	The production rate of the resistant type non-infected CD4^+^ T-cells per unit time.
*χ*_*w*_	The rate at which the wild type CD4^+^ T-cells are infected by the HIV virions.
*χ*_*r*_	The rate at which the resistant CD4^+^ T-cells are infected by the HIV virions.
*μ*_*T*_*w*__	The death rate of the wild type CD4^+^ T-cells.
*μ*_*T*_*r*__	The death rate of the resistant type CD4^+^ T-cells.
*μ*_*I*_	The death rate of the infected CD4^+^ T-cells.
*ϵ*_*V*_	The number of virions releases per bursting infected cells.
*μ*_*V*_	The death rate of the infectious virus.
*α*	The rate at which the infected cells are eliminated by the activated CTL T-cells.
*c*	Proliferation rate of CTL T-cells.
*λ*_*Z*_	The rate at which the cytotoxic T-cells are produced.
*μ*_*Z*_	The death rate of the CTL T-cells.
*β*	The rate at which the CTL T-cells are activated due to the presence infected CD4^+^ T-cells.
*μ*_*Z*_*a*__	The rate at which the activated defense cells decay.

**Table 2:** Sensitivity indices of *R*_0_ evaluated at the baseline parameter.

Parameters	Parameter value	sensitivity index
*λ*_*T*_*w*__	10	0.428724544
*λ*_*T*_*r*__	0.03198	0.571275455
*χ*_*T*_*w*__	0.000024	0.428724544
*χ*_*T*_*r*__	0.01	0.571275455
*μ*_*T*_*w*__	0.01	−0.428724544
*μ*_*T*_*r*__	0.01	−0.571275455
*ϵ*_*V*_	100	1
*μ*_*V*_	3	−1

**Table 3:** Parameters for *in vivo* HIV model.

Parameters	Value	Source
*λ*_*T*_*w*__	10 cell/mm^3^/day	Attarian and Tran [2]
*λ*_*T*_*r*__	0.03198 cell/mm^3^/day	Attarian and Tran [2]
*μ*_*T*_*w*__	0.01 day^−1^	Srivastava et al. [14]
*μ*_*T*_*r*__	0.01 day^−1^	Attarian and Tran [2]
*χ*_*w*_	0.000024 mm^3^ vir^−1^ day^−1^	Attarian and Tran [2]
*χ*_*r*_	0.01 mm^3^ vir^−1^ day^−1^	Attarian and Tran [2]
*μ*_*I*_	0.5 day^−1^	Wodarz and Nowak [7]
*ε*_*V*_	100 vir. cell^−1^ day^−1^	Mbogo et al. [15]
*μ*_*V*_	3 day^−1^	Mbogo et al. [15].
*α*	0.02 day^−1^	Arruda et al. [9]
*λ*_*Z*_	20 cell/mm^3^/day	Arruda et al. [9].
*μ*_*Z*_	0.06 day^−1^	Arruda et al. [9]
*c*	0.000005 L^2^ cells^2^ day^1^	Zarei et al. [12]
*β*	0.004 day^−1^	Arruda et al. [9]
*μ*_*Z*_*a*__	0.004 day^−1^	Arruda et al. [9]
